# RETRACTION: Potential Therapeutic Role of Hispidulin in Gastric Cancer Through Induction of Apoptosis via NAG-1 Signaling

**DOI:** 10.1155/ecam/9823616

**Published:** 2025-06-12

**Authors:** Evidence-Based Complementary and Alternative Medicine

RETRACTION: C. Y. Yu, K.-Y. Su, P.-L. Lee, et al., “Potential Therapeutic Role of Hispidulin in Gastric Cancer Through Induction of Apoptosis via NAG-1 Signaling,” *Evidence-Based Complementary and Alternative Medicine* 2013 (2013), https://doi.org/10.1155/2013/518301.

The above article, published online on 15 September 2013 in Wiley Online Library (https://wileyonlinelibrary.com), has been retracted by John Wiley & Sons Ltd.

The retraction has been agreed following the investigation of concerns raised by *Actinopolyspora biskrensis* on PubPeer [1]. The investigation has identified similarities between the Procaspase-3 and Cleaved-caspase-3 bands in Figure 2d, and the COX-2 and P65 bands in Figure 4a.

As a result of the investigation, the data and conclusions of this article are considered unreliable.

The authors have been informed of our decision to retract the article but did not provide a response.

## References

[1] https://pubpeer.com/publications/EE9865310C1DAF6DA11B9EE2CA2D7A#1.

